# P04-05 Testing muscle strength and dynamic balance in older recreational golfers and healthy sedentary non-golfers in community settings

**DOI:** 10.1093/eurpub/ckac095.059

**Published:** 2022-08-29

**Authors:** David A Wilson, Simon Brown, Paul E Muckelt, Martin B Warner, Sandra Agyapong-Badu, Roger A Hawkes, Andrew D Murray, Maria Stokes

**Affiliations:** 1 School of Health Sciences, University of Southampton, Southampton, United Kingdom; 2 Department of Health and Care Professions, University of Winchester, Winchester, United Kingdom; 3 Centre for Sport, Exercise and Osteoarthritis Research Versus Arthritis, Nottingham, United Kingdom; 4 School of Sport, Exercise and Rehabilitation Sciences, University of Birmingham, Birmingham, United Kingdom; 5 European Tour Performance Institute, Virginia Water, United Kingdom; 6 Physical Activity for Health Research Centre, University of Edinburgh, Edinburgh, United Kingdom; 7 School of Health Sciences, University of Southampton, Southampton, United Kingdom

**Keywords:** Ageing, physical activity, golf, strength, balance

## Abstract

**Background:**

Regular physical activity is known to reduce premature mortality, and help prevent and manage chronic diseases. Despite this, older people are not sufficiently active. Playing golf is associated with better aerobic fitness and mental wellbeing but evidence of a relationship with strength and balance is lacking. If the physical demands of golf are sufficient to meet the World Health Organisation recommendations for strength and balance, golf may qualify for exercise on prescription/social prescribing for people with long-term conditions. The hypothesis of this ongoing study is that playing recreational golf will be associated with better strength and balance in older people. Data are presented for grip strength and dynamic balance in golfers and sedentary older adults, tested using simple techniques suitable in community settings.

**Methods:**

Seventy nine healthy older participants (aged 65-79 years) have been studied: 62 golfers (n = 31 females, 31 males) and 17 sedentary non golfers (9 males, 8 females). Difficulties in recruiting sedentary participants and then the outbreak of Covid-19 explain the discrepancy between group sizes. Golfers played 18 holes at least once a week for minimum of two years. Grip strength was tested for the right hand using the MIE hand-grip dynamometer, with results normalized to body weight. Dynamic balance was assessed using the Y-balance test, with reaching distance normalized to lower-limb length. Non-parametric statistics were used due to unequal group sizes.

**Results:**

Grip strength was significantly greater in golfers than non-golfers (median and interquartile range); males and females combined; golfers 4.3±1.2; non-golfers 3.3±1.9 (*p*=0.039*; Mann-Whitney). The Y-balance performance was also significantly better in golfers than non-golfers (*p*=0.002*: Mann-Whitney). Normalised composite reach distance data (3 directions) for the right side were greater in golfers (81.7±13.3) than non-golfers (74.2±17.2).

**Conclusions:**

These preliminary data indicate that playing recreational golf at least once a week is associated with greater grip strength and better dynamic balance in older golfers compared to sedentary non-golfers. These findings support further data collection (when permitted) to produce reference data. This will allow parametric statistical analysis to determine whether conclusive evidence will support the hypothesis, forming the basis of a randomised controlled trial.

